# Development of lymphocyte subpopulations in local breed chickens

**DOI:** 10.14202/vetworld.2021.1846-1852

**Published:** 2021-07-19

**Authors:** Adil Sabr Al-Ogaili, Samer Sadeq Hameed

**Affiliations:** 1 Department of Medical Laboratory Techniques, Kut Technical Institute, Middle Technical University, Baghdad, Iraq; 2 Department of Pathology and Poultry Diseases, College of Veterinary Medicine, University of Baghdad, Baghdad, Iraq

**Keywords:** direct immunofluorescent staining, flow cytometry, local breed chickens, lymphocytes subpopulations

## Abstract

**Background and Aim:**

Local breeds of chicken are known to have relatively higher disease resistance to many endemic diseases and diseases that are highly virulent in commercial chickens. This study aimed to address the lymphocyte subpopulations in three constitutive immune system organs (thymus, bursa of Fabricius, and spleen) in 30, 8-week-old, male local breed chickens.

**Materials and Methods:**

The T (CD3^+^) and B lymphocytes (Bu-1^+^) were identified through one-color, direct immunofluorescent staining of the thymus, bursa, and spleen lymphocytes. Likewise, two-color, direct immunofluorescent staining was performed to identify the CD4- and/or CD8-defined T lymphocytes. The proportions of T and B lymphocytes and CD4- and/or CD8 defined chicken lymphocyte subsets in lymphoid suspensions prepared from the thymus, bursa, and spleen were determined by flow cytometry.

**Results:**

CD3^+^ cells, particularly those positive for CD4^+^CD8^–^, were dominant in the thymus, whereas cells expressing the Bu-1 marker were predominant in the bursa of Fabricius. The proportion of T and B cells was almost equal in the spleen, with more cells expressing the CD4^–^CD8^+^ marker in the red pulp.

**Conclusion:**

These findings indicate that local breeds of chicken could serve as a reliable model for studying the immune system of commercial light chicken breeds, due to the similarity in the presence and the distribution of the immune cells.

## Introduction

Lymphocytes are responsible for acquired immune responses in higher vertebrates. As in other vertebrates, the lymphocytes in chickens are divided into B and T lymphocytes, depending on their origin and function [1,2]. Both cell lineages are further subdivided into distinct subpopulations depending on the presence of specific markers on the cell surface [3]. However, the traditional methods for the examination and differentiation of these cells are not sufficient to recognize the major and minor subpopulations. At present, molecular-based methods appear to be most accurate to distinguish between the cell types [4]. In chickens and many domesticated avian species, Bu-1 is a constant B lymphocyte marker (in pre-plasma B cells) before maturation [5,6]. On the other hand, T lymphocytes express CD3^+^ complexes (T cell receptor). Subsets of T lymphocytes express additional surface markers such as CD4, a common marker of T helper lymphocytes (T_H_), whereas CD8 is the common surface marker of cytotoxic T lymphocytes [7,8]. In the thymus, the precursor thymocyte population expresses CD4^–^CD8^–^, which later develops into CD4^+^CD8^–^ or CD4^–^CD8^+^ T lymphocytes. These single positive T lymphocytes respond specifically to certain antigens. The CD4^+^CD8^–^ cell, better known as CD4 cell, is responsible for inducing an immune response against exogenous antigens. On the other hand, the CD4^–^CD8^+^ cell, also known as CD8 cell, is responsible for orchestrating an immune response against exogenous antigens [9,10]. Tag-labeled specific monoclonal antibodies are used to distinguish these cell markers [11].

Local breeds of chickens are more refractory to many highly pathogenic diseases that cause serious outbreaks among commercial poultry [12]. Yet, published reports on the development of a lymphocyte subpopulation in these breeds are scarce.

In this study, we sought to determine the ­presence of any shift in the density and distribution of professional immune cells in a local breed of male chickens at the molecular level. The presence and density of T and B lymphocytes in 8-week-old local breed male chickens were examined by determining the specific surface markers through immunohistochemistry and flow cytometry.

## Materials and Methods

### Ethical approval

The study and all tests and procedures were approved by the Scientific and Animal Care Committee, Department of Pathology and Poultry Disease, College of Veterinary Medicine, University of Baghdad.

### Study period and location

This study was conducted from March to June 2020. Fertile eggs hatchery were kept in and the hatched birds were raised in the premises of Kut-Tech Institute, Middle Technical University, Kut, Wasit, Iraq.

### Chickens

Thirty, 8-week-old male local breed chickens were used. Only male chickens were selected to exclude the effect of sex on the immunity of these birds. The breeding process was started by incubating the eggs from local breeds in a hatchery using the 1-day sex protocol. The birds were raised under sterile conditions with water and feed *ad libitum*; no vaccines or antibiotics were used. At the age of 8 weeks, the birds were euthanized and the immune system organs were collected under sterile conditions.

### Weights and weight percentages of the birds and their primary and secondary lymphoid organs

The chickens were euthanized and weighed. The primary lymphoid organs (thymus and bursae) and spleens were dissected and weighed. The average of the total weights of the lymphoid organs and their percentages in relation to the average of the total bodyweight were calculated as previously described [13].

### Preparation of single cell suspension from the spleen, thymus, and bursa of Fabricius

The splenocytes were separated by density gradient centrifugation using the Ficoll-Paque method (Fico/Lite LymphoH™, Atlanta Biologicals, USA; density 1.077). Slices of the spleen (about 0.2-0.25 g/organ/bird) stored in icy 1× phosphate-buffered saline (1×PBS; pH 7.4). These slices were passed through a nylon mesh (pore size, 60 mm; Tetko, Elmsford, NY) and were collected in a sterile beaker This was followed by the immediate addition of cold 1×PBS to cover the tissue pieces. The contents of the beaker were transferred to a sterile, screw-capped tube. The splenocyte suspension was washed by spinning at 250×*g*, at 4°C for 8 min. The supernatant fluid was discarded, and the pellet was re-suspended in 5 mL of 1×PBS at room temperature (RT). Next, 5 mL of the spleen cell suspension was carefully layered over 5 mL of the Ficoll 1077 at RT. The mixture was centrifuged at RT, 400×*g* for 30 min. After centrifugation, the cells at the PBS/Ficoll interface were aspirated using a Pasteur pipette and placed into a tube containing 8 mL of cold 1×PBS [14-16]. The splenocytes were washed 3× by centrifugation at 250×*g* and 4°C, for 8 min. The pellet formed was re-suspended in 2 mL of cold 1×PBS and placed on ice.

The 1×PBS was treated with 0.1% sodium azide to prevent the cells from internalizing the markers and labels; 1% bovine serum albumin was used to block and prevents the non-specific binding of the antibodies [14].

As with the spleens, the bursae and thymi were dissected and weighed. One thymic lobe and a piece of bursa of Fabricius were collected from each bird (~0.2 g/organ/bird). The samples were cut into pieces and stored in 1×PBS on ice. The tissue pieces were forced through a nylon mesh as described earlier. Cold 1×PBS was immediately added until the tissue pieces were covered by the solution. However, the thymocyte cell suspension has a high fat content; therefore, fat was completely removed from the suspension after the third wash (by centrifugation as described for the splenocytes). The pellet was re-suspended in 5 mL of cold 1×PBS. After washing the cells again, the supernatant was discarded, and the pellet was re-suspended in 5 mL of ice-cold 1×PBS [17-19].

### Determination of the cell concentrations

The concentrations of the splenocytes, thymocytes, and bursa cells were determined using a hemocytometer (stage-objective, 40×). Then, 20 μL of the cell suspension was added to 180 μL of Trypan blue-PBS (0.04% w/v in 1×PBS) in a microcentrifuge tube and mixed well. The stain penetrates dead cells and stains the proteins blue. The cells were diluted with 1×PBS until a final concentration of 4×10^7^ cell/mL was reached [20].

### Immunohistochemistry

Frozen sections of spleen (thickness, 6 mm) were obtained using a cryostat (temperature, −22°C) (Thermo Fisher Scientific, USA). The sections were fixed in acetone for 5 min using poly-L lysine-coated slides (Sigma-Aldrich). Inside a humidifying chamber, the tissues were stored in PBS/10% horse serum ([HS] to prevent non-specific staining) (Thermo Fisher Scientific) overnight at RT. After incubation and three washes with 1×PBS, 80 μL of a primary antibody/diluent was added and the sections were incubated for 30 min at RT. The sections were washed again and 80 μL of biotinylated horse anti-mouse immunoglobulin (Ig) G was added as the secondary antibody (Thermo Fisher Scientific).

The sections were incubated for 30 min at RT followed by 5 washes with PBS. Then, 80 μL of avidin-biotin complex reagent was immediately added and the sections were incubated for 30 min at RT. The sections were washed (5 times) and 100 μL of “charged” DAB (3, 3’-diaminobenzidine) (Abcam, USA) was added to each slide for color development. After a final course of washing, methyl green was added to the sections, which were then incubated for 1 h. The slides were dipped in tap water and passed through a series of dehydrating baths of ethanol as follows: 70%, 95%, and 100% for 30 s, 100% ethanol-100% Americlear (50:50 mix) for 15 s, and 100% Americlear for 1 min [21,22].

### Flow cytometry

Cell suspensions from primary and secondary lymphoid organs, that is, thymus, bursa of Fabricius, and spleen, were subjected to flow cytometry procedure. For the one-color, direct immunofluorescent staining procedure, mouse anti-chicken ­CD3-fluorescein isothiocyanate (FITC)-conjugated monoclonal antibody (mouse IgG1) (Southern Biotech, Alabama, USA), and mouse anti-chicken Bu-1-phycoerythrin (PE)-conjugated mAb (mouse IgG1) (Southern Biotech) were used to determine the percentages of T (CD3^+^) and B (Bu-1^+^) cells in the three cell suspensions, respectively. Alternatively, mouse anti-chicken CD4-FITC-conjugated mAb (mouse IgG1) (Southern Biotech) and mouse anti-chicken CD8-PE-conjugated mAb (mouse IgG1) (Southern Biotech) were used in the two-color, direct immunofluorescent staining procedure to identify the CD4^+^ and/or CD8^+^ markers, respectively, on the T lymphocytes.

Fifty microliters of each cell suspension (2 10^6^ cells) were added to a 96-well round-bottom microtiter plate (four columns were used/organ). The first column was used as the isotype control with FITC- and PE-conjugated mouse IgG1 that does not express any chicken molecules specificity (Sigma). Fifty microliters each of mouse anti-chicken CD3-FITC mAb and mouse anti-Bu-1-PE mAb were added to the second and third columns, respectively. A mixture (50 μL) of mouse anti-CD4-FITC and mouse anti-chicken CD8-PE mAb (ratio 1:1) was added to the fourth column. The procedure was conducted as previously described [3,4].

## Results

### Weight and weight percentage of the organs

The total bodyweights of the chickens and the weights of the primary and secondary lymphoid organs were measured. The average bodyweight was 1128 g. The proportion of each lymphoid organ was calculated depending on the individual organ in relation to the total bodyweight (Table-1).

**Table-1:** Measurement of the total weight and percentage of bodyweight of the thymus, bursa of Fabricius and spleen in 8-week old males local breed of chickens*.

Measurement^†^	Thymus	Bursa	Spleen
Weight (g)	4.107±0.292	3.64±0.14	2.48±0.206
% of BW^⧫^	0.213±0.009	0.322±0.007	0.131±0.012

Average of organ weights were calculated in grams and weight percentages.

* Bodyweight-adjusted lymphoid organs weight.

† Data correspond to the arithmetic means±SE of the results (n=30).

⧫ The average bodyweight (BW/gm) 1128±46.2

### Viable/dead cells in the primary and secondary lymphoid organs

The average percentages of viable and dead cells per gram of tissue and the percentages of viable and dead cells in relation to the weight of the whole organ are shown in Table-2.

**Table-2:** Number of viable cells per gram of tissue or per the whole organ.

Measurement^*^	Thymus	Bursa	Spleen
Cells/gram^†^Cells/organ^†^Average dead cells (%)^†^	1.25×10^9^4.59×10^9^19	2.22×10^9^3.89×10^9^14.9	6.27×10^8^1.53×10^9^29.7

The percentage of dead cells in thymus, bursa of Fabricius and spleen has been calculated in 8 week-old male local breed chickens,

* Cell suspension was prepared differently according to the organ evaluated. In case of spleen, density gradient centrifugation over Ficoll was performed. Thymocytes suspension did not require further purification steps.

† Average was taken after two readings in two chambers of the hemocytometer

### Immunohistochemistry

#### Thymus and bursa of Fabricius

**Figure-1:**
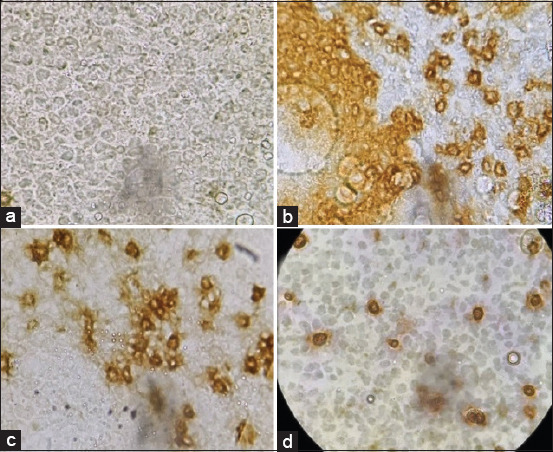
Thymus and bursa of Fabricius sections showing (a) isotype control. (b) Bursa of Fabricius cell showing lymphocytes expressing Bu-1 marker (brown in color). (c) Thymocytes expressing CD4 marker (brown-stained cells). (d) Thymocytes expressing CD8 marker (brown-stained cells). Primary antibody and biotinylated horse anti-mouse IgG secondary antibody were the first to treat the section. A ”charged” DAB (Abcam, USA) was the primary stain to develop color and methyl green was the counterstain.

Sections from the thymus showed dense cell populations expressing both CD4 and CD8 markers (Figure-1c and d). On the other hand, the bursa of Fabricius cells demonstrated dense populations of lymphocytes, which expressed the Bu-1 marker (Figure-1b).

#### Spleen

**Figure-2:**
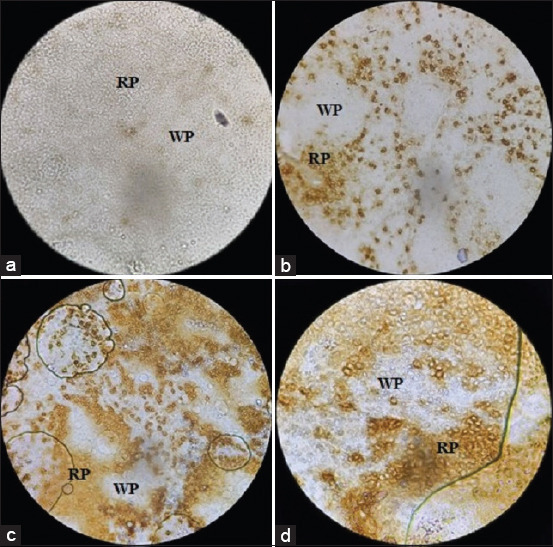
Spleen section showing: (a) Isotype control. (b) CD8 (CD4^–^CD8^+^) lymphocyte stained in brown color especially in red bulb (RP) 40×. (c) Lymphocytes expressing CD3 marker 100×. (d) Lymphocytes expressing Bu-1 marker 100×. Primary antibody and biotinylated horse anti-mouse IgG secondary antibody were the first to treat the section. A ”charged” DAB (Abcam, USA) was the primary stain to develop color and methyl green was the counterstain.

Lymphocytes with CD8 (CD4^–^CD8^+^) marker were dominant in the red bulb of the spleen (Figure-2b). Other lymphocytes expressing, the CD3 (Figure-2c) and Bu-1 (Figure-2d) markers were detected over scattered batches within the red and white bulbs of the spleen.

### Flow cytometry

**Figure-3:**
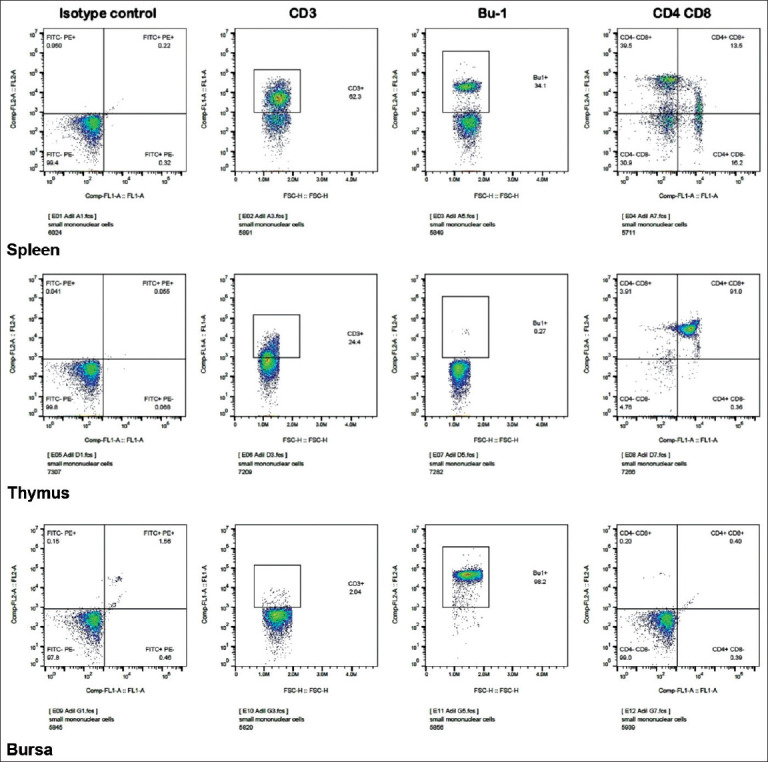
Flow cytometry results of cell suspension from spleen, thymus, and bursa of Fabricius. T lymphocytes (CD8 in particular with CD4^–^CD8^+^ marker) were the dominant cells in the spleen. Thymocytes show that the CD3 marker is denser than that in bursa. Conversely, the Bu-1 marker is highly expressed by the lymphocytes in bursa. All the lymphocytes in the thymus were expressing both CD4 and CD8 markers (CD4^+^CD8^+^) with traces of CD4^–^CD8^-^, CD4^+^CD8^–^, and CD4^–^CD8^+^. In the bursa, almost all the lymphocytes are expressing no marker (CD4^–^CD8^+^).

The data obtained showed that T lymphocytes (particularly those with the CD8 marker) were dominant in the spleen (Figure-3/spleen). Thymocytes showed that the CD3 marker was the denser marker expressed among all the cells in thymus. Moreover, all the lymphocytes in the thymus expressed both CD4 and CD8 (CD4^+^CD8^+^) with traces of CD4^–^CD8^-^, CD4^+^CD8^–^, and CD4^–^CD8^+^ (Figure-3/thymus). Conversely, the Bu-1 marker was highly expressed by the lymphocytes in the bursa (Figure-3/bursa). However, the vast majority of the lymphocyte population in the bursa was not expressing any marker (i.e. CD4^–^CD8^–^). Thus, various lymphocyte subpopulations were identified within each lymphoid organ using both flow cytometry and immunohistochemistry. These two procedures, i.e., flow cytometry and immunohistochemistry, have been applied to cell suspension obtained from the lymphoid organs (Table-3).

**Table-3:** Determination of lymphocyte population subsets in lymphoid organs.

Cell phenotype	Thymus (%)	Spleen (%)	Bursa of Fabricius (%)
CD3^+^ (T-Cell)	24.4	62.3	2.04
Bu-1^+^ (B-Cell)	0.27	34.1	98.2
CD4^+^CD8^-^ (T-helper)	0.36	16.2	0.39
CD4^-^CD8^+^ (Cytotoxic T-Cell)	3.91	39.5	0.2
CD4^+^CD8^+^	91	13.5	0.4
CD4^-^CD8^-*^	4.76	30.9	99
Total CD4^+**†**^	91.36	29.7	0.79
Total CD8^+**‡**^	94.91	53	0.6
Small mononuclear cells**^¦^**	73.1	60.2	58.4

Flow cytometry procedure has been applied for cell suspensions from thymus, bursa of Fabricius and spleen. Depending on the lymphocyte cell markers, the proportion of each cell was calculated in 8-week-old local breed male chickens (n=30).

* Very immature thymocytes,

† includes CD4^+^CD8^-^ and CD4^+^CD8^+^,

‡ includes CD4^-^CD8^+^and CD4^+^CD8

## Discussion

Several attempts are being made to understand the immune cells and immune pathways in commercial chickens due to the economic importance of these breeds [7,23]. However, it is equally important to understand the immune regulation system in local breeds of chicken, particularly in developing countries. Indigenous breeds of chickens stand in an intermediate position between wild and commercial breeds [24].

Despite being cosmopolitan, rural or local breeds of chickens show significant refraction for most infections [25,26]. In the present study, we sought to determine the presence and density of the acquired immune system cells, that is, lymphocytes and their populations, in the primary and peripheral lymphoid organs. The lymphocyte viability was observed and the viable cells were counted within the lymphoid organs. The data showed similarity to those in genetically divergent breeds of chicken (Table-1). In addition, the weights and weight percentage of the primary and secondary lymphoid organs were within the normal range when compared to those of their commercial counterparts (light breeds in particular) (Table-2) [27]. Like other vertebrates, avian lymphocytes are distributed in the primary lymphoid organs; they migrate to the secondary lymphoid organs where they encounter the antigen. The lymphocytes are divided into B and T lymphocytes, based on their sites of origin and maturation [28]. In the present study, T and B lymphocytes were detected in the thymus with the balance shifted in favor of the T lymphocytes. Furthermore, CD3^+^ cells were dominant in the thymus and spleen (Figures-1 and 3 and Table-3). In the bursa of Fabricius, lymphocytes expressing the Bu-1 marker were predominant (Figures-1 and 3). Around 98% of the lymphocyte population in this primary lymphoid organ expressed the Bu-1 marker (Table-3). In the spleen, the proportion of the two cells was almost equally distributed with more cells expressing the CD4^–^CD8^+^ marker in the red pulp (Figure-2). This could be attributed to the main function of the spleen as a secondary immune organ. The T lymphocytes encounter their cognate antigen-presenting cells in the spleen. In addition, B lymphocytes have the tendency to relocate over the zones according to their activation status [29-31].

Analysis of the lymphocyte subpopulations in the thymus revealed that all the various markers (CD4^–^CD8^–^, CD4^+^CD8^–^, CD4^–^CD8^+^, and CD4^+^CD8^+^) were expressed by the cells. However, the proportion of each cell type was different (Figure-3). Thymocytes with CD4^+^CD8^–^ markers were predominant, whereas the marker CD4^+^CD8^+^ was the least marker expressed by these cells. This is consistent with the findings of Bucy *et al*. [30]. The CD4^–^CD8^–^ marker was highly expressed in the bursa (Figures-1 and 3). On the other hand, the spleen, which is a secondary lymphoid organ, harbored dense populations of both T and B lymphocytes, with a relative dominance of T lymphocyte (particularly the CD8 type of cells), which might be attributed to the fact that the CD8 cytotoxic T cells encounter the endogenous antigens (Figure-3 and Table-3) [27,32]. However, these proportions are changeable due to several factors, including the presence of an infection.

## Conclusion

Our results showed that despite genetic divergence, local breeds of chicken could serve as a good and reliable model for studying the immune system of commercial breeds of chicken due to similarities in the presence and distribution of the immune cells [33,34]. No significant variations in the presence of these immune cells and their distribution within the primary or secondary immune organs have been recorded when compared to their commercial counterparts (commercial light breeds in particular) [35]. Therefore, there is no detectable immune variability in favor of the local breed when confronted by infections. The natural relative resistance of these breeds could be attributed to hygiene practices. Therefore, these birds might have undergone long-term natural selection [36].

## Authors’ Contributions

ASA and SSH: Conceived and designed the experiment, conducted and analyzed the data, and contributed to the manuscript drafting and revisions. Both authors read and approved the final manuscript.

## References

[1] Litman G.W, Rast J.P, Fugmann S.D (2010). The origins of the adaptive immunity. Nat. Rev. Immunol.

[2] Júnior A.F, dos Santos J.P, Sousa I.O, Martin I, Alves E.G.L, Rosado I.R (2018). *Gallus gallus* domesticus:Immune system and its potential for generation of immunobiologics. Ciência Rural.

[3] Fair J.M, Taylor-McCabe K.J, Shou Y, Marrone B.L (2008). Immunophenotyping of chicken peripheral blood lymphocyte subpopulations:Individual variability and repeatability. Vet. Immunol. Immunopathol.

[4] De Boever S, Croubels S, Demeyere K, Lambrecht B, De Backer P, Meyer E (2010). Flow cytometric differentiation of avian leukocytes and analysis of their intracellular cytokine expression. Avian Pathol.

[5] Weber W.T (2000). *In vitro* characterization of chB6 positive and negative cells from early avian embryos. Cell. Immunol.

[6] Igyarto B.Z, Nagy N, Magyar A, Olah I (2008). Identification of the avian B-cell-specific Bu-1 alloantigen by a novel monoclonal antibody. Poult. Sci.

[7] Bridle B.W, Julian R, Shewen P.E, Vaillancourt J, Kaushik A.K (2006). T lymphocyte subpopulations in commercially raised chickens. Can. J. Vet. Res.

[8] Lee I.K, Gu M.J, Ko K.H, Bae S, Kim G, Jin G, Kim E.B, Kong Y, Park T.S, Park B, Jung H.J, Han S.H, Yun C (2018). Regulation of CD4^+^CD8^?^CD25^+^ and CD4^+^CD8^+^CD25^+^ T cells by gut microbiota in chicken. Sci. Rep.

[9] Chen C.H, Göbel T.W.F, Kubota T, Cooper M.D (1994). T cell development in the chicken. Poult. Sci.

[10] Sekelova Z, Polansky O, Stepanova H, Fedr R, Faldynova M, Rychlik I, Vlasatikova L (2017). Different roles of CD4, CD8 and gd T?lymphocytes in naive and vaccinated chickens during *Salmonella enteritidis* infection. Proteomics.

[11] Williamson S.L.H, Steward M, Milton I, Parr A, Piggott N.H, Krajewski A.S, Angus B, Horne C.H.W (1998). New monoclonal antibodies to the T cell antigens Cd4 and CD8. Am. J. Pathol.

[12] Msoffe P.L.M, Mtambo M. M. A, Minga U.M, Gwakisa P.S, Mdegela R.H, and Olsen J.E (2002). Productivity and natural disease resistance potential of free-ranging local chicken ecotypes in Tanzania. Livest. Res. Rural Dev.

[13] M'Sadeq S.A, Wu S, Choct M, Swick R.A (2018). Influence of trace mineral sources on broiler performance, lymphoid organ weights, apparent digestibility, and bone mineralization. Poult. Sci.

[14] Bewrger C.L, Edelson R.L (1979). Comparison of lymphocyte functions after isolation by Ficoll-hypaque floatation and elutriation. J. Invest. Dermatol.

[15] Wu Y, Lu H, Cai J, He X, Hu Y, Zhao H, Wang X (2009). Membrane surface nanostructures and adhesion property of T lymphocytes exploited by AFM. Nanoscale Res. Lett.

[16] Jergović M, Nedeljković G, Cvetić Ž, Gottstein Ž, Bendelja K (2017). Combined dextran and ficoll separation yields pure populations of chicken peripheral blood mononuclear cells-short communication. Vet. Arhiv.

[17] Mansour I, Bourin P, Rouger P, Doinel C (1990). A rapid technique for lymphocyte preparation prior to two-color immunofluorescence analysis of lymphocyte subsets using flow cytometry comparison with density gradient separation. J. Immunol. Methods.

[18] Rimondi1 A, Pinto S, Oliver V, Dibárbora M, Pérez-Filgueira M, Craig M.I, Pereda A (2014). Comparative and histopathological and immunological study of two field strains of chicken anemia virus. Vet. Res.

[19] Ko K.H, Lee I.K, Kim G, Gu M.J, Kim H.Y, Park B.C, Park T.S, Han S.H, Yun C (2018). Changes in bursal B cells in chicken during embryonic development and early life after hatching. Sci. Rep.

[20] Dulwich K.L, Asfor A.S, Gray A.G, Nair V, Broadbent A.J (2018). An *ex vivo* chicken primary bursal-cell culture model to study infectious bursal disease virus pathogenesis. J. Vis. Exp.

[21] Pertile T.L, Walser M.M, Sharma J.M, Shivers J.L (1996). Immunohistochemical detection of lymphocyte subpopulations in the tarsal joints of chicken with experimental viral arthritis. Vet. Pathol.

[22] Zhang Q, Waqas Y, Yang P, Sun X, Liu Y, Ahmed N, Chen B, Li Q, Hu L, Huang Y, Chen H, Hu B, Chen Q (2017). Cytological study on the regulation of lymphocyte homing in the chicken spleen during LPS stimulation. Oncotarget.

[23] Noujaim J.C, Filho R.L.A, Lima E.T, Okamoto A.S (2009). Detection of CD4+and CD8+lymphocytes in the intestine of broiler chicks treated with *Lactobacillus* spp. and challenged with *Salmonella enterica* serovar Enteritidis. Brazi. J. Poul. Sci.

[24] Hata A, Nunome M, Suwanasopee T, Duengkae P, Chaiwatana S, Chamchumroon W, Suzuki T, Koonawootrittriron S, Matsuda Y, Srikulnath K (2021). Origin and evolutionary history of domestic chickens inferred from a large population study of Thai red junglefowl and indigenous chickens. Sci. Rep.

[25] Ikpeme E.V, Ekerette E.E, Efienokwu J.N, Ozoje M.O (2019). Immune response of Nigerian chicken genotypes to salmonella and Newcastle vaccines. Trends Appl. Sci. Res.

[26] Haunshi S, Rajkumar U (2020). Native chicken production in India:Present status and challenges. Livest. Res. Rural Dev.

[27] Hofman T, Schmuker S (2021). Characterization of chicken leukocyte subsets from lymphatic tissue by flow cytometry. J. Quant. Cell Sci.

[28] Birhan M (2019). Systemic review on avian immune systems. J. Life Sci. Biomed.

[29] Bucy R.P, Chen C.L, Cihak J, Lösch U, Cooper M.D (1988). Avian T cells expressing gamma delta receptors localize in the splenic sinusoids and the intestinal epithelium. J. Immunol.

[30] Bucy R.P, Chen C.L, Cooper M.D (1990). Ontogeny of T cell receptors in the chicken thymus. J. Immunol.

[31] Lewis S.M, Williams A, Eisenbarth S.C (2019). Structure-function of the immune system in the spleen. Sci. Immunol.

[32] Jeurissen S.H, Janse E.M, Ekino S, Nieuwenhuis P, Koch G, de Boer G.F (1988). Monoclonal antibodies as probes for defining cellular subsets in the bone marrow, thymus, bursa of Fabricius, and spleen of the chicken. Vet. Immunol. Immunopathol.

[33] Hammer D.K (1974). The immune system in chickens. Avian Pathol.

[34] Mpenda F.N, Schilling M.A, Campbell Z, Mngumi E.B, Buz J (2019). The genetic diversity of local African chickens:A potential for selection of chickens resistant to viral infections. J. Appl. Poult. Res.

[35] Psifidi A, Banos G, Matika O, Desta T.T, Bettridge J, Hume D.A, Dessie T, Christley R, Wigley P, Hanotte O, Kaiser P (2016). Genome-wide association studies of immune, disease and production traits in indigenous chicken ecotypes. Genet. Sel. Evol.

[36] Banos G, Lindsay V, Desta T.T, Bettridge J, Sanchez-Molano E, Vallejo-Trujillo A, Matika O, Dessie T, Wigley P, Christley R.M, Kaiser P, Hanotte O, Psifidi A (2020). Integrating genetic and genomic analyses of combined health data across ecotypes to improve disease resistance in indigenous African chickens. Front. Genet.

